# *EXTENSIN18* is required for full male fertility as well as normal vegetative growth in *Arabidopsis*

**DOI:** 10.3389/fpls.2015.00553

**Published:** 2015-07-22

**Authors:** Pratibha Choudhary, Prasenjit Saha, Tui Ray, Yuhong Tang, David Yang, Maura C. Cannon

**Affiliations:** ^1^Department of Biochemistry and Molecular Biology, University of Massachusetts Amherst, AmherstMA, USA; ^2^Plant Biology Division, The Samuel Roberts Noble Foundation, ArdmoreOK, USA

**Keywords:** cell wall polymers, extensins, hydroxyproline-rich glycoproteins (HRGPs), gene expression, pollen, biomass

## Abstract

EXTENSINS (EXTs) are a 65-member subfamily of hydroxyproline-rich glycoproteins (HRGPs) of which 20 putatively form crosslinking networks in the cell wall. These 20 classical EXTs are involved at the start of new wall assembly as evidenced by a requirement for EXT3 during cytokinesis, and the ability of some EXTs to polymerize *in vitro* into dendritic patterns. EXT3 was previously shown to form pulcherosine (three Tyrosines) cross-links. Little direct data exists on the other 19 classical EXTs. Here, we describe the phenotypes of *ext18* mutants and rescued progeny as well as associated expression profiles of all 20 classical *EXT* genes. We found that *EXT18* is required for full male fertility, as well as for normal vegetative growth. EXT18 has potential to form crosslinking networks *via* di-iso-di-tyrosine (four Tyrosines) covalent bonds, and not *via* pulcherosine due to deficit of lone Tyrosines. This together with *ext18* defective pollen grains and pollen tubes, and reduced plant size, suggests that EXT18-type EXTs are important contributors to wall integrity, in pollen and other rapidly extending walls. The data also show that a knockout of *EXT18* had a pleiotropic affect on the expression of several *EXTs*, as did the reintroduction of the native *EXT18* gene, thus supporting the thesis that transcription of groups of *EXTs* are co-regulated and work in different combinations to make distinctive inputs into wall assembly of different cell types. These insights contribute to basic knowledge of cell wall self-assembly in different cell types, and potentially enable biotechnological advances in biomass increase and plant fertility control.

## Introduction

The outermost layer of young plant cells is a primary wall made up mainly of interpenetrating polymers of cellulose, hemicellulose, and pectins. Walls also contain proteins and glycoproteins with enzymatic and/or structural roles, most of unknown, or putative function based on sequence (Bacic et al., 1988; Fry, 2004; Albersheim et al., 2011). This dynamic matrix gives each cell, structure, shape, tensile strength, and protection, and consequently it is intimately involved in plant size and architecture. More recent technologies including Next Generation Sequencing, proteomics and fluorescence microscopy, have enabled the identification of molecular family members of wall components (Lum et al., 2013), and where and when they are located in the wall (Hruz et al., 2008). The ever-changing composition of growing walls, and their recalcitrance to examination have presented challenges to identifying wall components and how they assemble and function (Burton et al., 2010). This is no less the case with hydroxyproline-rich glycoproteins (HRGPs), a large family in relatively low abundance when compared to other wall components, and defined by the presence of hydroxyproline, glycosylated amino acid residues and repetitive glycomodules (Kieliszewski and Lamport, 1994; Seifert and Roberts, 2007; Showalter et al., 2010). HRGPs are associated with the early stage embryo (Ruiz-Avila et al., 1992; Hall and Cannon, 2002), and soybean root nodule development (Cassab, 1986), as well as mechanical stress (Tire et al., 1994; Shirsat et al., 1996), wound response (Showalter et al., 1991; Bradley et al., 1992), and defense (Esquerre-Tugaye and Lamport, 1979). Due to its multifarious associations with plant growth and development and plant defense mechanisms, a signaling role has frequently been attributed to HRGPs (Ellis et al., 2010), although a receptor has not been identified. More recent evidence supports a role for HRGPs as a component of global calcium signaling in plants (Lamport and Varnai, 2013).

The focus of the work presented here is a subfamily of HRGPs, the EXTENSINS (EXTs), initially identified over 50 years ago (Lamport, 1963; Chen and Varner, 1985), and now considered to be structural components of primary cell walls (Cannon et al., 2008; Lamport et al., 2011). The *Arabidopsis* genome encodes 65 EXTs in total (Showalter et al., 2010); they are developmentally expressed (Hruz et al., 2008) and locate to the wall. EXTs are distinguished from other HRGP by the presence of the repetitive motif, Ser(Pro)_3-5_ (Lamport, 1973), where the Pro (P) is usually Hydroxyproline (Hyp, O) and arabinosylated, and the Ser (S) galactosylated. These hydrophilic carbohydrate motifs contribute to stabilizing EXTs in an extended conformation (Stafstrom and Staehelin, 1986) and are critical for function as demonstrated by the pleiotropic phenotypes associated with loss-of-function of Hyp *O*-arabinosyltransferase (*HPAT*) genes coincident with under-arabinosylation of EXT3 (Ogawa-Ohnishi et al., 2013).

There are 20 ‘classical EXTs’; the remaining 45 are hybrids with non-EXT HRGPs, or chimeras with non-HRGP domains (Johnson et al., 2003). A typical classical EXT is identified by its alternating hydrophilic and hydrophobic amino acid motifs composed of a higher than usual content of a few amino acids arranged in a periodic sequence (Smith et al., 1986; Memelink et al., 1993): usually an abundance of Tyr (Y) alone and/or as Y-X-Y, which may form isodityrosine (Idt; Fry, 1982), and a positively charged amino acid residue, Lys (K) or occasionally His (H), at regular intervals (Kieliszewski and Lamport, 1994). EXT sequences predict a polyproline II rod shaped structure (van Holst and Varner, 1984).

The amino acids, motifs and glycomodules of EXTs and their strict periodicity are important because they provide the capacity for these molecules to self-assemble based on hydrophilic/hydrophobic attractions, i.e., like-with-like (Ohno, 1994), followed by covalent crosslinking to form di-Idt (has four Y; Brady et al., 1996), and purcherosine (has three Y; Brady et al., 1998), catalyzed by extensin peroxidase (Schnabelrauch et al., 1996). Staggered overlapping EXTs forming dentritic shaped networks have been observed by AFM for several purified EXTs (Cannon et al., 2008; Lamport et al., 2011). These positively charged scaffolds are hypothesized to allow an EXT network to provide a template on which acidic pectins can be organized during cell wall assembly. This ‘EXT self-assembly model’ is supported by the observed effect of purified EXT on pectin gel formation: non-covalent interaction occurred supporting a role for basic amino acid residues of EXT interacting with pectin (MacDougall et al., 2001). The model is further supported by examination of multilayered thin films formed by EXT and pectin using a layer-by-layer method (Decher, 1997; Valentin et al., 2010).

Different combinations of *EXT* genes are expressed in different tissue and cell types through out the growth of *Arabidopsis* (Hruz et al., 2008), and some EXTs have been localized to growing cell walls using antibodies to associated glycan epitopes (Swords and Staehelin, 1993; Casero et al., 1998) and gene tags (Hall and Cannon, 2002). Finding the locations and functional significance of specific EXTs has been a greater challenge, due to the sequence homology within this large gene family and possible functional overlap. A loss of function has been reported for a knock-down of EXT3, however, the complexity of assigning function and determining mechanism of action of EXTs is exemplified by the discovery that an alternative gene expression program involving several cell wall genes alleviates a requirement for EXT3 (Saha et al., 2013). A root hair phenotype is associated with knocking out the Leu-rich-repeat (LRR) hybrid EXT, LRX1 (Baumberger et al., 2001) and its paralog LRX2 (Baumberger et al., 2003). Knocking out the EXT domain alone also produces the mutant phenotype, suggesting that the function of the EXT domain is the tethering of the signaling domain, likely to the EXT network. Short and/or irregular root hair lengths were shown in plants with insert mutations in *EXT6, 7, 10, 11, 12*, and *13*; all showed haploid insufficiency, while plants homozygous for the insert showed more severe phenotypes (Velasquez et al., 2011). Root hair cell wall phenotypes are also associated with mutated prolyl-4-hydroxylases (P4H), required for the hydroxylation of Pro to Hyp (Tiainen et al., 2005), indicating a requirement for HRGP(s) in root hair growth (Velasquez et al., 2011).

In the *ext* mutants described so far, the *EXTs* in question are in the walls of rapidly growing cells, i.e., embryo, elongating root and root hair cells. Pollen tubes also have rapidly growing cell walls (Hepler et al., 2013), and several *EXT* genes are expressed in growing pollen tubes of *Arabidopsis* (Hruz et al., 2008), and a chimeric *EXT* gene was shown to be expressed in maize almost 20 years ago (Rubinstein et al., 1995). The detection methods used in these pollen studies were based on EXT probes, primers, and antibodies that could not discriminate between individual EXT family members. Therefore, a specific network forming classical EXT had not been identified in pollen. The approach taken here using EXT18 specific primers, was to identify and confirm two *ext18* alleles and to examine both their vegetative and reproductive phenotypes, as well as to examine expression of all 20 *EXT* genes, using discriminating primers, in one of the alleles and its rescued progeny. We provide evidence in support of a role for EXT18 (At1g26250), a classical EXT, in pollen grain wall integrity and successful pollen tube growth, as well as in vegetative growth and biomass yield.

## Results

### The *ext18* Insert Lines Confirmed and Mutant Phenotype Demonstrated

**FIGURE 1:**
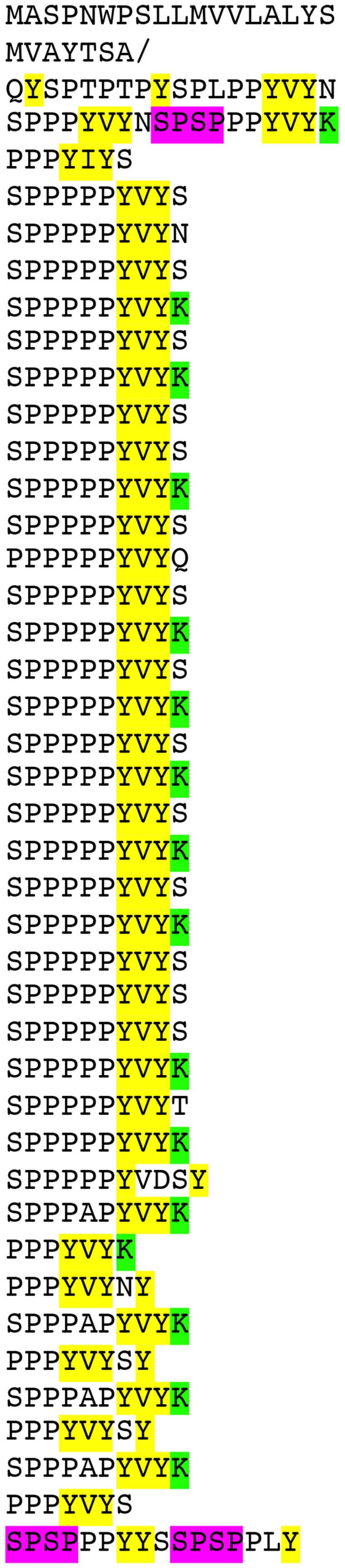
**Predicted amino acid sequence of WT non-hydroxylated pre-EXT18 (At1g26250), arranged to emphasize the 10-residue major repetitive motif (MRM).** Like other classical EXTs, EXT18 has an N-terminal sequence and a C-terminal sequence containing mainly the same amino acid residues found in the MRM but not exactly in register. Amino acids considered relevant to function are: YELLOW, highlights the terminal Y, lone Y, and 2Y residues, and putative Isodityrosine (Idt) motifs; GREEN, highlights the positively charged amino acids; MAGENTA, highlights SPSP motifs. Note the deficit of lone Y residues compared to EXT3 (Supplementary Figure S3). **/**, indicates cleavage site of the 25 residue predicted signal peptide (Bendtsen et al., 2004).

**FIGURE 2:**
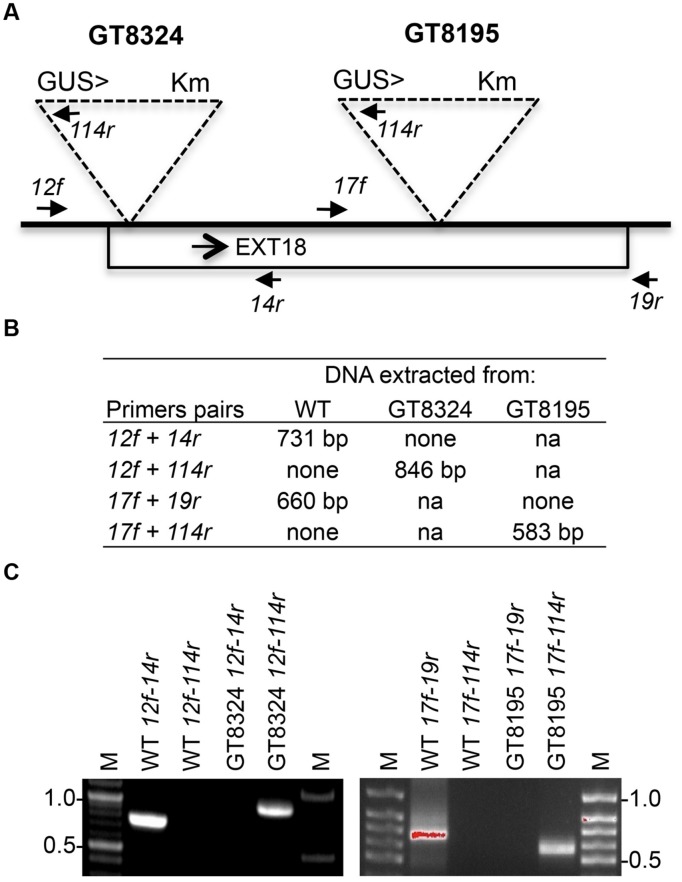
**Strategy and confirmation of *EXT18* (At1g26250) insert lines. (A)** Map (not to scale) of the *EXT18* gene, showing direction (arrow), gene-trap inserts GT8324 and GT8195, primers (closed head arrows) used to confirm insert locations, the *NPTII* gene (for Kanamycin resistance, Km), and the *UidA* gene (for the GUS assay) and its direction (>) with respect to *EXT18*. **(B)** Primer pairs used and band sizes obtained in base pairs (bp) by PCR. **(C)** Amplicons separated by 1% agarose gel electrophoresis. Images allow comparison of band sizes from WT DNA with those of homozygous insert lines GT8324 and GT8105 following amplification with the primers indicated. For WT DNA the bands show: (i) the expected native DNA fragment sizes obtained using native primers for PCR, (ii) the absence of these native DNA fragment sizes when one of the primer pairs matches to the insert. For the insert lines the bands show: (i) the absence of native DNA fragment sizes using native primers for PCR, (ii) the fragment sizes obtained using one native primer and one insert primer. M, molecular size markers in kilobase pairs (kb).

Insert lines GT8324 and GT8195, where the EXT18 peptide-coding region (**Figure 1**) is interrupted by an insert, were identified and confirmed (**Figure 2**). PCR analysis placed the gene trap inserts of GT8324 and GT8195 following amino acid positions ∼23 and ∼290, respectively, in the 443 residue pre-protein. Also PCR analysis showed that the β-glucuronidase (GUS) gene in both insert lines was in the same orientation as that of *EXT18*. For each of the insert lines, starting with individual heterozygous plants (labeled F1), segregation analyzes of progeny, following self-fertilization, showed inheritance of the insert that did not differ significantly from the Mendelian ratio of ∼3:1 on kanamycin (km) plates (km resistant:km sensitive), indicating that these gene-trap lines each had an insert at one location only.

**FIGURE 3:**
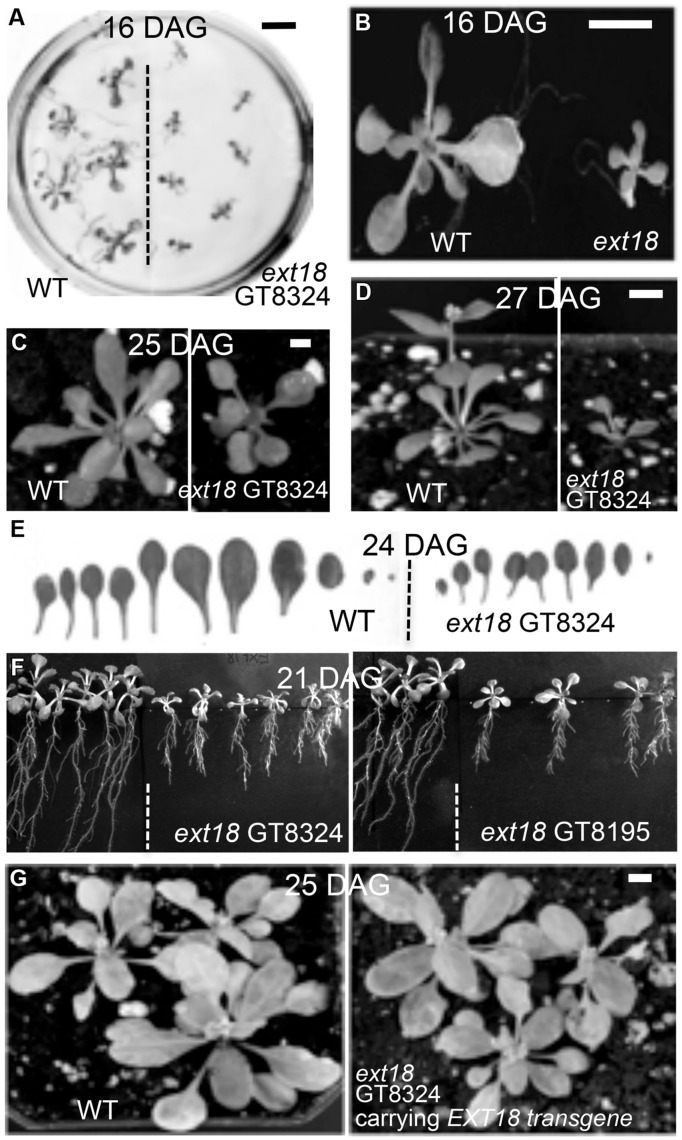
**Reduced vegetative growth and delayed flowering time of homozygous *ext18* compared to WT, and mutant phenotype rescue by the native *EXT18* transgene. (A)** 16 DAG seedlings of WT (left) and *ext18* GT8324 (right) germinated on half-MS medium. Mutant seedlings are smaller than WT. **(B)** Magnification of two seedlings from **(A)**. **(C)** A typical WT (left) and *ext18* GT8324 (right) growing in soil at 25 DAG. Bolting is visible at rosette center of WT, but not in *ext18*. **(D)** A typical WT (left) and *ext18* GT8324 (right) growing in soil at 27 DAG. The bolt is more advanced in WT. **(E)** Rosette leaves collected from a typical 24 DAG WT (left) and *ext18* GT8324 (right). Mutant leaves are fewer and smaller than WT. **(F)** WT (left of dashed lines) and *ext18* insert lines as indicated following vertical incubation of plates for 21 days. **(G)** Three typical WT (left) and three typical homozygous *ext18* GT8324 expressing an *EXT18* transgene (right), at 25 DAG. No visible differences in vegetative growth or flowering times were observed. Bars = 1 cm **(A,B)**; 0.5 cm **(C,D,G)**.

**FIGURE 4:**
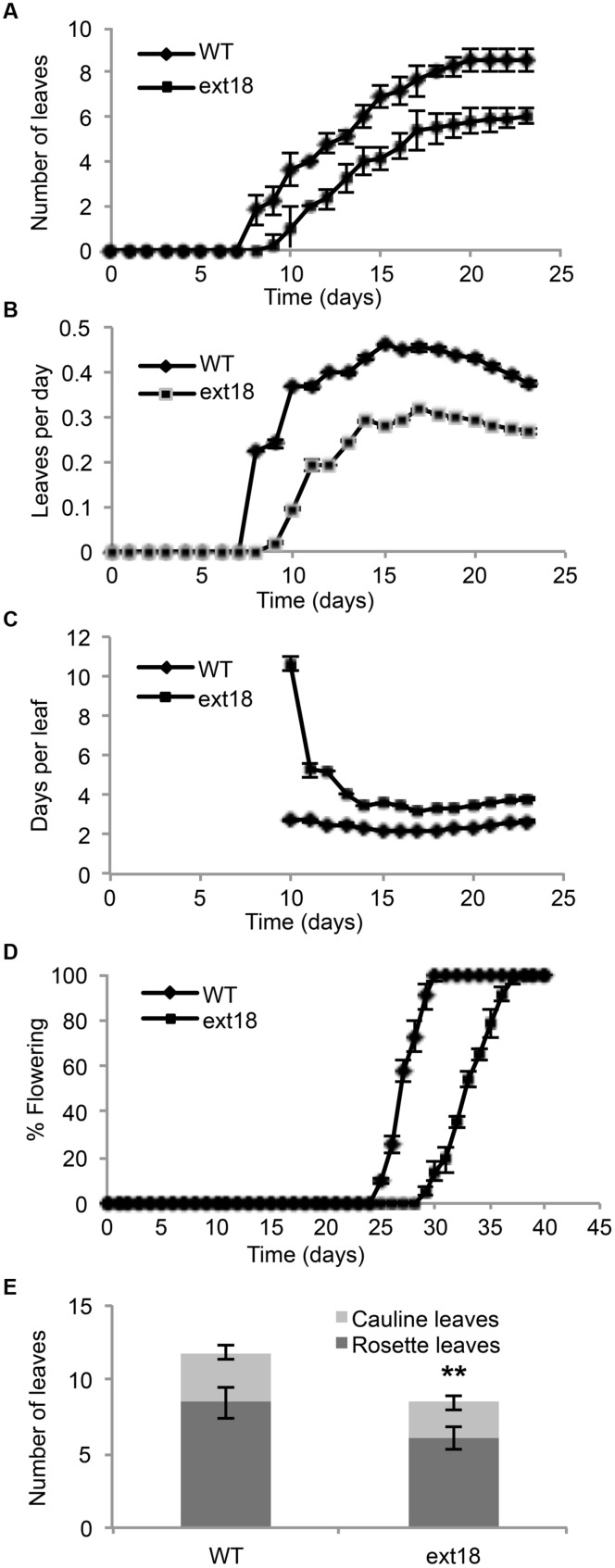
**Vegetative and reproductive growth of WT and *ext18* GT8324 under long-day conditions. (A)** Number of leaves as a function of time, from leaf bud to flower bud. Values are from three independent experiments, *n* = 40 (WT) and 57 (*ext18*). **(B)** Rate of leaf production (leaves per day) from germination to appearance of flower bud. Values are from four independent experiments, *n* = 100 (WT) and 150 (*ext18*). Rate of leaf production is slower in *ext18*, consequently the plastochron (days/leaves) in **(C)** is prolonged. **(C)** Rate of leaf production in days per leaf, i.e., the plastochron. **(D)** Percent of flowering as a function of time. Flowering time was taken when flower bud was visible. Values are from three independent experiments of *n* = 40 (WT) and 57 (*ext18*). **(E)** Total number of rosette and cauline leaves per plant. Values are from three independent experiments, *n* = 40 (WT) and 57 (*ext18*). ^∗∗^Numbers of both rosette and cauline leaves in *ext18* compared to the WT were significantly different (*P* values of 9.94E-18, and 1.68E-12, respectively). Error bars in all panels = SD.

Phenotypes of the insert lines, both heterozygotes and homozygotes, were compared with their wild-type progenitor (WT) showing that the mutations were recessive and affected both vegetative and reproductive plant parts. In-depth visual examinations were carried out with both *ext18* homozygous insert lines, confirming reduced growth, from seedling to flowering compared to WT (**Figures 3A–F**). Both insert lines showed similar phenotypes to each other and different to that of WT at all stages of growth. A quantification of seed germination and roots showed that the *ext18* insert lines had significantly lower seed germination frequencies, and shorter roots with more lateral roots than WT (**Table 1**). Quantification of vegetative growth and flowering initiation, (as described Méndez-Vigo et al., 2010) of *ext18* GT8324 showed a reduced plastochron (PL, leaf initiation rate) compared to WT throughout vegetative growth (**Figures 4A–C**), and a delayed flowering time (**Figure 4D**). The *ext18* mutant plants produced significantly fewer total leaves (6.05 ± 0.77 rosette leaves and 2.37 ± 0.53 cauline leaves) than WT (8.45 ± 1.08 rosette leaves and 3.3 ± 0.46 cauline leaves; **Figure 4E**). To the nearest whole day, 57% of the WT population flowered in 27 days, whereas 54% of *ext18* mutant population flowered at day 33 (FT_50_, defined as the days after germination (DAG) taken to flower by 50% of the plant population in this study). The fewer leaves and longer time to flowering indicate a delayed plastochron in the *ext18* mutant. These data suggest a delayed flowering time because of slower organ initiation rate, and not because of delayed ‘transition to flowering.’

**Table 1:** Quantification of seed germination and root morphology of WT and *ext18* insert lines.

Phenotypes	WT Mean ± SD	GT8324 Mean ± SD	GT8195 Mean ± SD
Seed germination at 7 days DAG (%)^a^	95.9 ± 1.1	41.2 ± 4.5	60.7 ± 1.5
Lateral roots per cm at 21 DAG^b^	1.6 ± 0.1	6.0 ± 0.4	4.8 ± 0.2
Root length in cm at 21 DAG^b^	5.8 ± 0.6	2.9 ± 0.6	4.4 ± 0.8

*^a^*n* = 1080, 1350, and 1071, respectively, for WT, GT8324 and GT8195; ^b^*n* = 48.*

*All comparisons of *ext18* with WT showed significant differences (*P* < 0.001).*

### The *ext18* Mutant has Reduced Fertility

**FIGURE 5:**
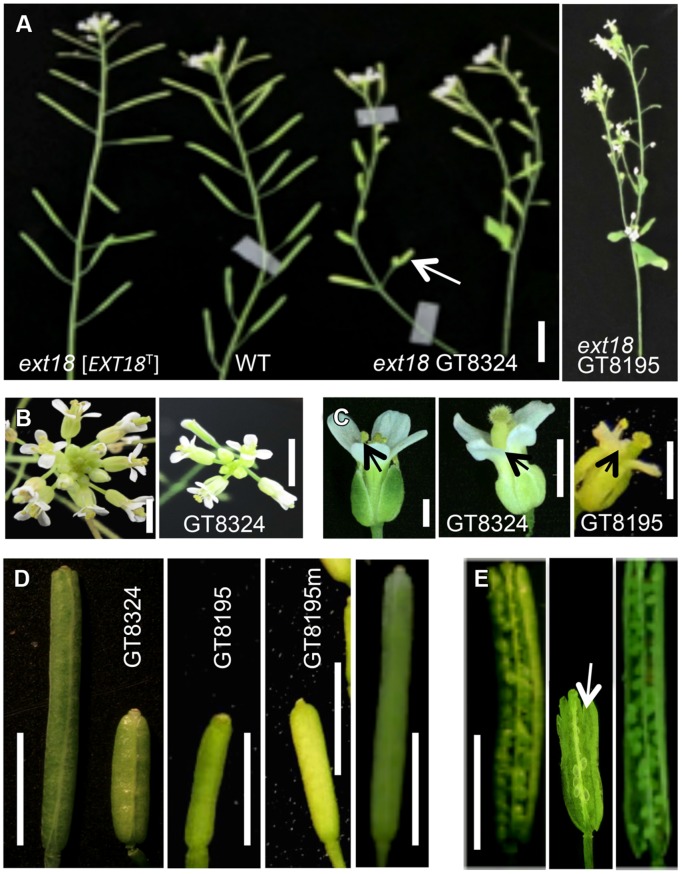
**Reproductive parts of *ext18* insert lines compared to WT. (A)** Upper part of main bolts of 35 DAG plants, as labeled. The *ext18* GT8324 line expressing an *EXT18* transgene [*EXT18*^T^] looks similar to WT while both *ext18* mutant lines have thinner bolts, fewer flowers and smaller siliques. Arrow indicates average size of the *ext18* mutant silique. Bar = 0.5 cm. **(B)** Typical inflorescence of WT (left) and *ext18* as indicated. The *ext18* insert lines produces fewer flower buds than WT. Bar = 5 mm. **(C)** WT (left), and *ext18* mutant flowers as indicated. Arrows point to the anthers. The *ext18* insert lines have smaller flowers and shorter anther filaments; most are hidden behind the petals. Bar = 1 mm. **(D)** Typical siliques of WT (left), insert lines as indicted, and *ext18* GT8324 [*EXT18*^T^] (right). The GT8195m is typical of siliques on more mature *ext18* plants. Bar = 0.5 cm. **(E)** An opened silique of a typical *ext18* GT8324 shows few developed seed (arrow) compared to that of WT (left) and *ext18* GT8324 [*EXT18*^T^] (right). Bar = 0.5 cm.

Both *ext18* insert lines had lower seed setting and seed yield compared to WT. An examination of the reproductive phenotype of the insert lines compared to WT, showed that both insert lines had shorter and thinner flower stems, produced fewer and smaller flowers, and had four or five identifiable anthers of varying lengths, most being shorter than the pistil; they also had shorter siliques, fewer seed per silique and a higher percentage of sterile seed (**Figure 5**; **Tables 1** and **2**). These data support a role for EXT18 in both plant growth and fertility. Plant quantification data combined (**Tables 1** and **2**; **Figures 3–5**) show that the overall biomass of *ext18* insert lines is considerably less than that of WT.

**Table 2:** Quantification of reproductive stages of growth of WT, *ext18* GT8324 and three rescued transgenic lines.

Phenotype	WT	*ext18* mutant^a^	*ext18* rescued line 1	*ext18* rescued line 2	*ext18* rescued line 3
Plant height (cm)^b^	27.24 ± 1.63	7.47 ± 1.46	16.89 ± 1.74	17.85 ± 1.32	17.3 ± 1.48
Stem width (cm)	0.73 ± 0.08	0.45 ± 0.08	0.62 ± 0.08	0.66 ± 0.08	0.62 ± 0.6
Opened flowers per plant^c^	85.2 ± 9.71	29.95 ± 4.99	83.9 ± 3.72	86.9 ± 1.19	84 ± 4.38
Unopened flowers per plant	0.23 ± 0.61	11.63 ± 4.32	2.0 ± 0.66	2.7 ± 0.48	2.6 ± 0.69
Siliques per plant^c^	85.2 ± 9.71	29.95 ± 4.99	83.9 ± 3.72	86.9 ± 1.19	84 ± 4.38
Sterile siliques (%)^c^	1.32 ± 1.04	28.20 ± 6.86	1.67 ± 0.63	1.49 ± 0.77	2.14 ± 0.92
Seeds per (fertilized) silique	41.09 ± 2.89	7.39 ± 1.26	37.8 ± 3.19	39.6 ± 2.59	40 ± 3.65
Unfertilized ovules per silique	2.0 ± 1.02	11.70 ± 2.39	1.3 ± 0.48	1.6 ± 0.69	1.3 ± 0.67
Total ovules per silique	43.09 ± 3.0	19.09 ± 2.34	39.1 ± 3.07	41.2 ± 2.74	41.3 ± 3.59
Silique length (mm)	11.34 ± 1.03	5.6 ± 1.1	10.1 ± 1.1	10.1 ± 1.1	10.5 ± 1.3

*^a^All *ext18* and WT comparisons showed significant differences (*P* ≤ 1.1E-18).*

*^b^Rescued lines had significantly less height than WT (*P* ≤ 1.9E-11).*

*^c^All open flowers produced siliques, but in *ext18*, 28% of siliques were sterile.*

*Mean values and SDs presented (WT and *ext18, n* = 41–43; rescued lines, *n* = 10–12).*

### Native *EXT18* Rescues the *ext18* Mutant Phenotype

To provide further evidence that a mutation in *EXT18* is associated with the observed mutant phenotype, the native *EXT18* gene, including 1,773 bp up-stream and 679 bp downstream of the amino acid coding region, was transformed into the *ext18* mutant lines to test for mutant rescue. Several independent transformants were identified from both insert lines, and with the exception of plant height all were visually indistinguishable from WT (**Figure 3G**). Quantification of phenotypic traits in three rescued lines of GT8324 confirmed the visual observations (**Table 2**; **Figures 5A,D,E**). Rescue by the *EXT18* transgene of the vegetative and reproductive mutant phenotypes of both the *ext18* insert lines provides further evidence that the mutant phenotypes of the two insert lines were caused by the inserts in the *EXT18* gene.

### *EXT18* Plays a Role in Male Fertility

**FIGURE 6:**
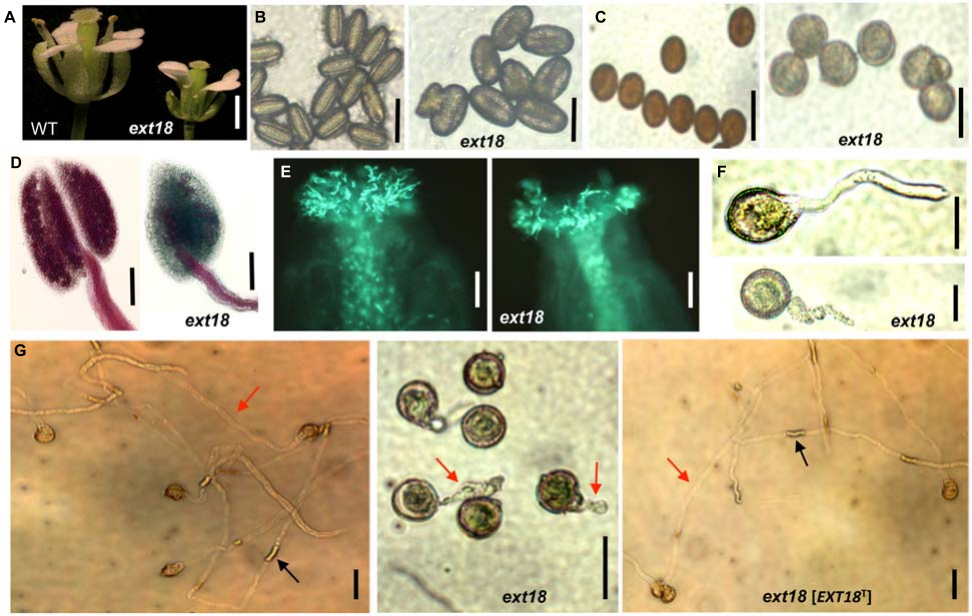
**Phenotypic analysis of *ext18* GT8324 pollen and pollen tube growth. (A)** Dissected flowers exposing the anthers from which pollen was tested. **(B)** Bright-field views of mature pollen from WT (left) and *ext18* (right*)*. **(C)** I_2_–KI-stained mature pollen from WT (left) and *ext18* (right*)*. **(D)** Alexander vitality staining of pollen from WT (left) and *ext18* (right). **(E)** *In vivo* pollen germination. Aniline blue-stained carpels of self-pollinated WT (left) and *ext18* (right). **(F)** *In vitro* pollen germination at ∼2 h of WT (upper), *ext18* (lower). **(G)** *In vitro* pollen germination at ∼8 h from WT (left), *ext18* (center), and *ext18* [*EXT18*^T^] (right). Red arrow = pollen tube; black arrow = callose plug. Bars = 1 mm **(A)**; 50 μm **(B,C,F,G)**; 250 μm **(D)**; 100 μm **(E)**.

**FIGURE 7:**
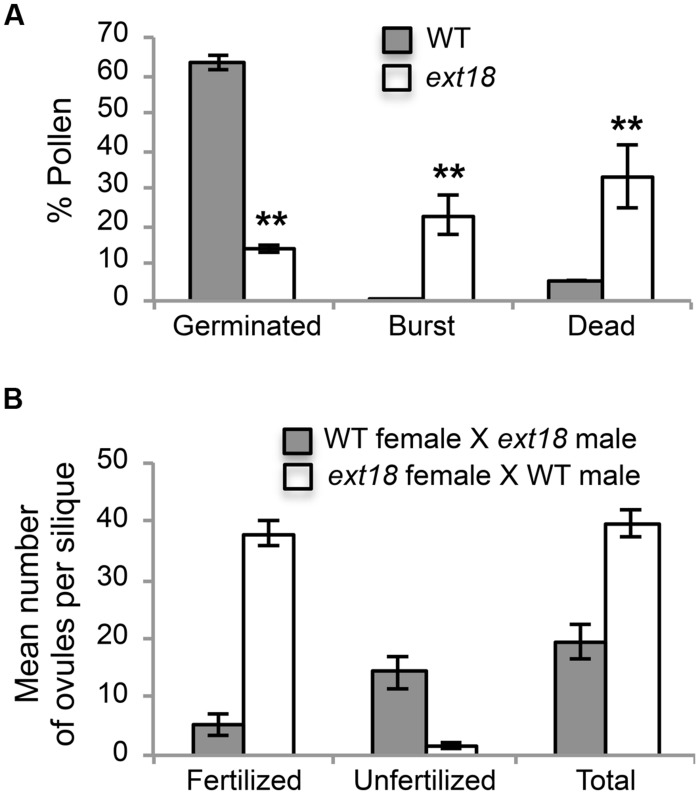
**Pollen viability and functionality quantified. (A)** Percentages of WT and *ext18* GT8324 pollen that were germinated, burst, or dead -obtained by counting each group following *in vitro* germination. Dead pollen was distinguished from live pollen following the Alexander Stain. Values are from three separate experiments with a total pollen count of *n* = 360 (WT) and 363 (*ext18*). **(B)** Functionality of pollen was quantified by reciprocal crossing of WT and *ext18* GT8324. Siliques from ten independently crossed flowers of WT and ten of *ext18* were dissected to count fertilized and unfertilized ovules and expressed as a mean number of ovules per silique. Differences with WT were significant (*P* ≤ 0.005). All error bars = SD.

Most, but not all, anthers in *ext18* flowers of both insert lines appeared abnormally short relative to the carpel, and most were hidden behind petals (**Figures 5C and 6A**). To investigate this, pollen from mature anthers was stained with DAPI to observe the nuclei: both the *ext18* mutant and WT showed normal two sperm nuclei and one vegetative nucleus, indicating that the mutant had normal meiosis. Having ruled that out as a possible cause for the *ext18* mutant phenotype, the general morphology of *ext18* GT8324 pollen was compared to WT in bright field microscopy: the results showed that *ext18* pollen is oval to round compared to the more rod shaped WT pollen, consistent with the mutant having weaker walls (**Figure 6B**). Images of I_2_–KI stained (Lugol solution) pollen showed also that the mutant pollen is larger and rounder, and less stained than WT indicating less storage starch in the mutant, which could result in reduced viability (**Figure 6C**). Pollen viability was examined using the Alexander stain, which distinguishes live (purple) from dead (green) pollen grains: the results showed less live pollen in the mutant compared to WT (**Figure 6D**). The percentage of dead to total pollen in each plant was counted, with *ext18* anthers showing 33% dead pollen compared to 6% for WT (**Figure 7A**). This lack of viable pollen as well as short anthers could be responsible for the low seed set found in *ext18* GT8324 and GT8195.

**FIGURE 8:**
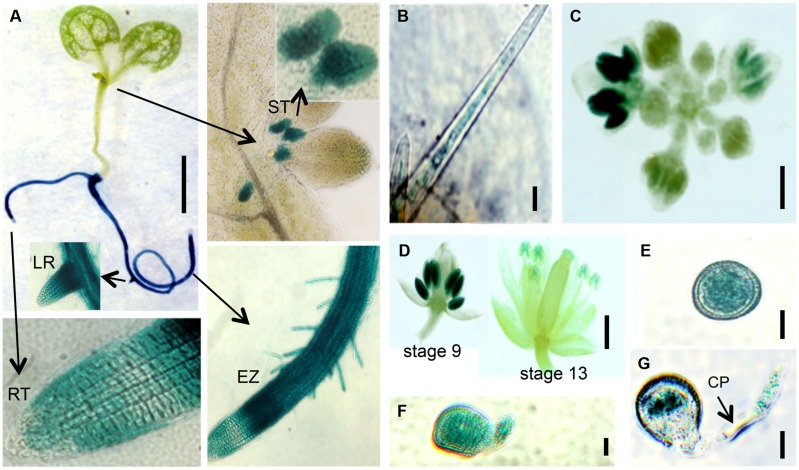
***EXT18* expression as determined by GUS reporter gene expression. (A)** A 10 DAG seedling shows GUS expression in roots and the stipule area. Higher magnification (arrows) shows expression in the stipules (ST), lateral root bud (LR), root elongation zone (EZ), and in root hairs, but not at the root tip (RT). **(B)** Expression in a leaf trichome, but not in the leaf. **(C)** Expression in the inflorescence. **(D)** Expression was most prominent in anthers from the 9th to the 13th stages of flowering. **(E)** Expression in pollen grains. **(F)** Expression in a pollen grain with a burst pollen tube (*ext18* GT8324). **(G)** Expression in a pollen grain and intact pollen tube. Note the unstained callous plug (CP). Bars = 1 mm **(A,C,D)**; 25 μm **(B,E,F,G)**.

Another contributing factor to low seed set in *ext18* mutants could be compromised pollen germination. A hint of this can be seen in the Aniline Blue image of carpels with germinating pollen (**Figure 6E**), where *ext18 in vivo* pollen germination appeared less than WT, and the carpels more hazy, which could be a consequence of burst pollen tubes with release of callose (Aniline Blue stains callose). To further examine the question of defective germination, pollen grains of the *ext18* mutants and WT were incubated on germination medium and observed for up to 10 h (**Figures 6F** and **8F,G**). A high number of pollen tube burst events were recorded for *ext18* compared to WT; only 14% of *ext18* pollen successfully germinated *in vitro* compared to 63% for WT (**Figure 7A**). This finding is in keeping with the reduced and more-hazy fluorescence seen in the *ext18* image of the Aniline Blue stained pollinated carpels (**Figure 6E**). These results are evidence that defective pollen tube growth as well as pollen viability are contributing factors to the *ext18* mutant having reduced seed set -a role that is supported by the fact that the *EXT18* transgene can restore viable pollen and pollen tube growth to the *ext18* mutant lines (**Figure 6G**).

**FIGURE 9:**
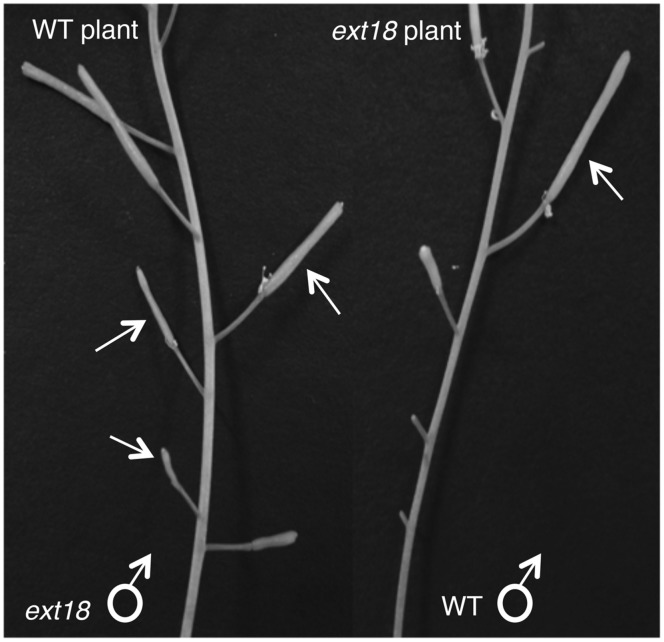
**Siliques from reciprocal crosses of WT and *ext18* plants.** The WT plant (left) when crossed with *ext18* pollen (arrows) produced small *ext18*-type siliques with reduced seed yield. The *ext18* plant (right) when crossed with WT pollen (arrow) produced normal looking siliques with WT level seed yield.

To further test the hypothesis that *EXT18* plays a role in male as distinct from female fertility, we did reciprocal crosses between the *ext18* and WT. When *ext18* pollen was used to fertilize WT, very few seed were produced and the siliques were short, while WT pollen fertilization of *ext18* had the opposite effect, i.e., full fertilization and full sized siliques were produced on *ext18* plants (**Figures 7B** and **9**; Supplementary Table S1). These data confirm that *ext18* makes defective pollen, and does not have a female gametophyte defect. The data also show that WT flowers fertilized with *ext18* pollen are unable to make full sized siliques, indicating that a full or some certain high level of pollination is required to produce normal sized siliques as well as seed yield.

### EXT18 Localizes to Expanding Cells, Pollen Grains, and Pollen Tubes

Localization of EXT18 was examined using the GUS reporter gene assay (**Figure 8**) made possible by the fact that the gene to detect GUS activity was in the same orientation as *EXT18* within its coding sequence. GUS activity was observed in the main root, lateral root, root hairs, and stipules. Activity was strongest in the elongation zones of the root, and lowest in cell division zone, and absent in the root cap. At all growth stages, roots continued to be GUS positive, while no GUS activity was observed in the inflorescence stem, rosette or cauline leaves. In developing flowers GUS activity was analyzed at various stages up to stage 18, when siliques started to loose their green color. Developing anthers from 9th to 13th stage of flowering showed strong GUS activity, as did pollen grains and tubes of germinating pollen. No GUS activity was observed in sepals, petals, and ovaries. Homozygotes and heterozygotes of *ext18* GT8324 and *ext18* GT8195 showed no difference in expression pattern. The GUS reporter gene analysis data show that EXT18 is developmentally regulated and is tissue specific. Relevant to the phenotypic studies of *ext18*, the GUS gene expression data show that EXT18 localizes to the sites most obviously defective in the *ext18* mutants.

### Knock-Out of *EXT18* has Pleiotropic Gene Expression Consequences

**FIGURE 10:**
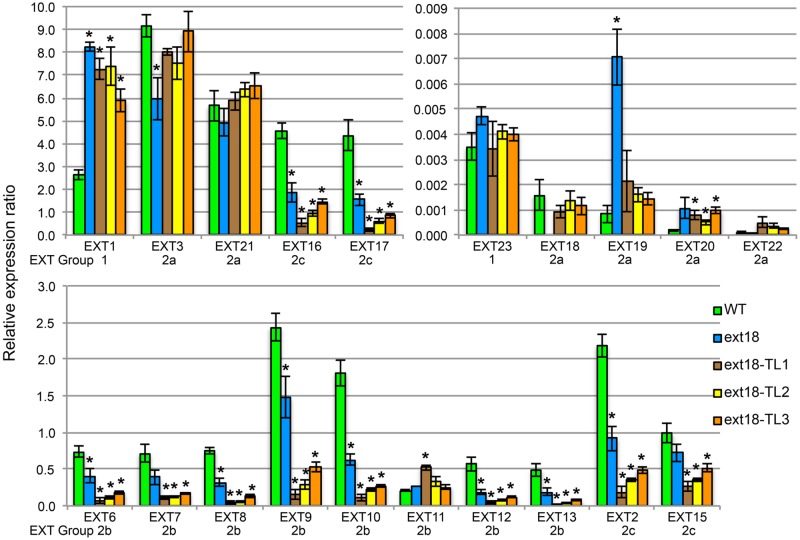
***EXT* gene expression analysis by qRT-PCR.** For purpose of presentation the 20 EXTs are presented in three sets of bar graphs based on the WT gene expression levels. Expression of each *EXT* is clustered for the five plant lines being compared: WT, the *ext18* GT8324 mutant, and three transgenic lines of *ext18* GT8324 that had been transformed with native *EXT18* gene (TL1, TL2, TL3). The classification group to which each EXT was previously assigned is named beneath each EXT cluster. ^∗^Expression significantly different to WT (*P* ≤ 0.05).

Expression of all twenty classical *EXT* genes was examined in seedlings of WT, the *ext18*-G8324 mutant, and three *ext18* G8324 lines transformed independently with the native *EXT18* gene. This more extensive analysis was carried out because earlier work showed that a change in expression of *EXT3* has pleiotropic effects on expression of other *EXT* genes (Saha et al., 2013). First and foremost here, the data show no detectable expression of *EXT18* in the *ext18* mutant, and restoration to approximately WT levels of *EXT18* expression in the three transgenic lines (**Figure 10**; Supplementary Table S2). These data support a requirement for *EXT18* in normal plant growth. An examination of all the expression data illuminates interesting points.

• Looking at *EXT* gene expression in the *ext18* mutant versus WT: expression of the other 19 *EXTs* in this mutant falls into three groups (*P* ≤ 0.05): (i) *EXTs* with up-regulated expression (*EXT1, -19*), (ii) *EXTs* with down-regulated expression (*EXT3, -6, -8, -9, -10, -12, -13, -2, -16, -17*), and (iii) *EXTs* with no change in expression. It is remarkable that *EXTs* with down-regulated expression compared to WT, except *EXT3*, are in EXT Groups 2b and 2c, i.e., the more complex EXTs.
• Looking at *EXT* gene expression in the three rescued *ext18* transgenic lines versus WT: it is remarkable that of the 12 *EXTs* that showed a significant difference in expression in the *ext18* mutant compared to WT, (apart from *EXT18* on transgene) only *EXT3* and *EXT19* were restored to WT levels (*P* ≤ 0.05) in the transgenic *ext18* lines.

## Discussion

The *ext18* insert lines GT8324 and GT8195 produce shorter roots and fewer and smaller leaves, flowers and siliques than WT; consequently they have a lower seed and overall biomass yield. Finding the leaf plastochron index as a method of measuring growth based on morphological measures rather than by chronological age showed that *ext18* plants had a slower leaf initiation rate and delayed flowering. The causes of reduced fertility are defects in pollen viability and pollen tube burst, also manifested as defective pollen grains and pollen tubes. These data suggest weak walls unfit for purpose due to the absence of EXT18. Reciprocal crosses of *ext18* and WT showed that the reproductive phenotype is exclusively a male reproductive problem. The GUS reporter gene assay showing EXT18 localizes to pollen grain and pollen tube, is in keeping with the defective pollen in the *ext18* mutants. Both insert lines showed the same mutant traits, and both lines were rescued, except for plant height (see *EXT1* discussion, below), by the *EXT18* transgene. This is strong evidence that mutations in *EXT18* are associated with the mutant phenotypes described and is supportive evidence that EXT18 plays a crucial role in cell wall integrity.

Given that the data support EXT18 involvement in male, and not in female reproduction, it is of interest to discuss why the segregation ratio of self-fertilized heterozygous *ext18* plants showed a ∼3:1 and not 2:1 (*ext18*:WT) as would be expected with 50% defective pollen in a heterozygote. However, *ext18* pollen is not fully defective, therefore the ratio would be expected to be higher than 2:1. How much higher would depend on what proportion of *ext18* pollen results in fertilization. To more fully understand this issue, a more detailed study of the pollen and its development in both homozygous and heterozygous *ext18* plants would be required. Here, we proceeded to focus on exploring EXT18 in the context of the other 19 EXTs.

A parallel between the *ext18* mutant phenotype and that of *hpat* loss-of-function mutants is mutually supportive evidence that one or more EXTs is required for normal vegetative and male reproductive genetic transmission, and that HPATs catalyze the arabinosylation of EXTs. The *hpat* under-arabinosylated mutants show pleiotropic phenotypic mutant traits including a decreased number of rosette leaves, and defects in cell wall thickening and pollen tube growth (Ogawa-Ohnishi et al., 2013). This comparative data further suggests that Hyp *O*-arabinosylation of EXTs is a prerequisite for assembly of stable EXT scaffolds.

Insights were gained as to the mechanism of EXTs function, by examining the expression profiles of all 20 classical *EXT* genes, and by considering how EXT18 relates to the ‘EXT self-assembly’ model (see Introduction). The absence of *EXT18* gene expression was shown to influence the level of expression of 12 other classical *EXT* genes – two were up-regulated and ten were down-regulated (*P* ≤ 0.05). This could be directly due to the absence of the *EXT18* transcript, or its translation product, or could be as a consequence of altered signaling, either directly, or indirectly due of a changed intracellular environment in *ext18* cells. Regardless of cause, it is clear that the absence of *EXT18* expression demonstrates co-regulation of combinations of *EXT* genes. Reintroduction of *EXT18* resulted in the restoration of only one (*EXT19*) of the two up-regulated genes (in *ext18*), and only one (*EXT3*) of the ten down-regulated genes, to WT expression levels, thereby demonstrating that alternative profiles of *EXT* gene expression, very likely alternative levels of different EXTs can produce apparently WT-phenotypes. The different gene expression profiles support the hypothesis that different members of this large EXT family build walls fit for purpose by involving the cross-linking of different EXTs within a polymeric network. If this were not the case, then a WT-phenotype could not be achieved using different combinations of EXT family members.

Could *EXT19*, the most up-regulated of the 20 *EXTs* in the *ext18* mutant, be partially compensating for the absence of EXT18? This seems a likely scenario given that both are Group 2a EXTs; EXT19 is the most closely related EXT to EXT18 having an 84% Similarity Index, and almost identical amino acid motifs within a major repetitive motif (MRM) of 10 amino acids (**Tables 1** and **3**; Supplementary Figure S1). Despite this, it is clear that EXT19 at best is only partially able to compensate for the absence of EXT18 (because *ext18* insert lines grow and produce some seed while having a mutant phenotypes). However, the results support the model that at least some EXTs, in this case EXT18, make specific, possibly unique contribution(s) to wall structure, and especially in the case of pollen as demonstrated here.

**Table 3:** Number of Amino acid residues and motifs relevant to function in EXT18, EXT19, EXT1, and EXT3.

EXT features	EXT18	EXT19	EXT1	EXT3
Signal peptide	25	26	23	27
Mature protein	418	452	354	404
MRM^a^ residues	10	10	7, 9	28
MRM is amphiphilic	yes	yes	yes	yes
YXY motifs	43	44	3	16
Lone Y residues	3	4	36	28
SPSP motifs	3	1	0	0
tri-C motifs	0	0	0	0
K, or/and H in MRMs	yes	yes	yes	yes

*^a^Major repetitive motive.*

Could the up-regulation of *EXT1* in the *ext18* mutant be a contributor to the mutant phenotype, or an aid to its survival, albeit enfeebled? Based on amino acid sequence analysis there is no reason to suspect either. EXT1 and EXT18 have a 52% Similarity Index, and are in different EXT groups (**Table 3**; Supplementary Figure S2). The question also arises from previously published work, where constitutive expression of *EXT1* resulted in thickening of *Arabidopsis* stems combined with reduction in plant height (Roberts and Shirsat, 2006). Evidence here weighs against EXT1 being a contributor to partial rescue because *ext18* mutant plants showed thin limp stems, and the *ext18*-transgenic lines did not have thicker stems than WT even though they had increased levels of *EXT1* expression. However, the up-regulation of *EXT1* in the *ext18*-transgenic lines may be responsible for reduced plant heights, as was the case with up-regulation of *EXT1* in previously published work (above). Since EXTs are known structural components of the cell wall, the data here further supports the hypothesis that at least some EXT family members play different and crucial roles in wall assembly and thereby in wall integrity. This is important because a distinctive role for individual EXT family members is unknown.

An association between individual EXTs, molecular assembly of EXT networks, and function, is very complex to decipher, but a comparison between what is known about EXT3, and now EXT18, allows us to begin comparing a pollen-EXT scaffold with a root-EXT scaffold in relation to the ‘EXT-self assembly model’ (see Introduction). Previous work shows that EXT3 is not detected in pollen, but is essential for WT plant growth (Hall and Cannon, 2002). EXT3 molecules form intermolecular crosslinks exclusively by pulcherosine (three Tyr; Cannon et al., 2008). Looking now at the MRMs of EXT18, it is clear that it has capacity to from only di-Idt (four-Tyr) intermolecular cross-links -unlike EXT3 there are no lone Tyr in EXT18 MRMs. EXT18 (and EXT19) has a MRM with a YXY motif (for crosslinking) at 10 amino acid residue intervals, while EXT3 has a 28 amino acid MRM each with a YXY (**Table 3**; Supplementary Figure S3). Of the EXTs with abundant YXYs in *Arabidopsis*, EXT18, EXT19, and EXT22 (At4g08380) are alone in having the shortest MRMs, while EXT3 is alone in having the longest MRM. This supports a model where a more covalently cross-linked EXT network with di-Idts as contributed by EXT18 could be appropriate for the pollen grain and rapidly growing pollen tube functions. This is evidence that different EXTs are involved in assembling walls fit-for-purpose in different cell types.

In the data presented here we show that *EXT18* makes a crucial contribution, either directly or indirectly, to pollen, and a less crucial albeit significant contribution to wall assembly in other plant parts. We provide evidence that relates phenotype/function to *EXT* gene expression and a requirement for EXT18. An obvious next step to consider is ultra-structural analysis of the mutant walls for the purpose of relating both function and gene expression to structure. However, given the pleiotropic effect of *EXT* gene knock-out on the expression of other *EXT* genes, differences in structure could be at best related to combinations and levels of EXTs. Interpreting such ultra-structure data would be further complicated by the fact that individual cells, even different walls of each cell type likely have different combinations of EXTs providing fractional contributions to the whole. The mutant *ext18* phenotype, including potentially observable wall structural changes could be directly due to the absence of EXT18 in the wall or it could be indirect, due to pleiotropic *EXT* gene expression. Pleiotropic *EXT* gene expression also explains why purified walls of *ext3* mutants showed no differences in amino acid content, including Pro, Hyp, and Tyr, i.e., the most abundant residues in EXTs (Saha et al., 2013). To further associate (i) *EXT* gene expression profiles, (ii) functionality, (iii) wall biochemistry, and (iv) structure, would require extensive analysis in all four areas using many plant parts in mutants and WT. Such research and analysis of the data arising is in the realm of ‘big data’ analysis.

## Materials and Methods

### Plant Lines, Growth Conditions, Segregation Analysis, and Plant Crosses

The gene-trap insertion lines GT8324 and GT8195 were obtained from Cold Spring Harbor Laboratory, NY (Sundaresan et al., 1995). These engineered transposon insert lines had been mapped to the amino acid coding sequence of EXT18 (At1g26250). Their progenitor *Arabidopsis thaliana* ecotype Landsberg *erecta* (WT) was obtained from Lehle Seed (Round Rock, TX, USA). Seeds were surface-sterilized with 70% ethanol for 60 s × 3, 90% ethanol for 90 s × 2, washed with sterile water × 3, and plated on medium containing half-strength MS salts and vitamins (Murashige and Skoog, 1962), 2% (w/v) sucrose and 0.6% agar, with pH adjusted to 5.7 (half-MS). Kanamycin 40 mg/liter (km^40^) was added to the medium after sterilization where mentioned. Seeds were stratified for 2 days at 4°C in the dark and then grown in a 16 h 140 μmol m^-2^ s^-1^ light/8 h dark cycle, at 22°C (long day conditions). Segregation analysis and/or transfer to soil were done at 12–14 DAG, which excludes the 2-days stratification. Reciprocal crosses were carried out by: picking apart the petals of unopened flowers, removing the stemens, and brushing the stigma with pollen from a donor flower.

### Identification of Insertion Sequences in *EXT18* Polymeric Lines

Genomic DNA was isolated using the DNeasy Plant Mini Kit (Qiagen, Germantown, MD, USA) and amplified in a thermocycler (MJ Research PTC-200) using 100 ng DNA, 10 μM dNTPs, 1 mM each of forward and reverse primers and 1 unit *Taq* polymerase in 20 μl reactions. The amplification program was: 5 min at 95°C, followed by 30 cycles of: 95°C for 1 min, 55°C for 1 min, 72°C for 60 s, and a final extension of 72°C for 10 min. Amplicons were separated by electrophoresis in a 1% agarose gel and images captured and recorded using Kodak Gel Logic 100 Imaging System. For strategy and primer sequences see **Figure 2** and Supplementary Table S3, respectively.

### Quantification of Seed Germination Frequencies and Seeding Root Morphology

To quantify seed germination, ½ MS plates with a total of 1080, 1350, and 1071 seeds from three different WT, GT8324 and GT8195 plants, respectively, were incubated horizontally, and results were taken at 7 DAG. To examine and quantify root morphology, plates were incubated vertically and results taken from a random 48 seedlings at 21 DAG. Significance for each comparison was calculated using the *t*-test and MS Excel 2010.

### Rate of Leaf Production, Plastochron, and Flowering Time

Based on published methods (Méndez-Vigo et al., 2010) the two reciprocal vegetative parameters, rate of leaf production (RLP, leaves per day) and plastochron (PL, days per leaf, also referred to as leaf initiation rate) were examined. While growing in a 16-h light/8-h dark regime, each plant was observed daily by the naked eye until flowering initiation. Leaf initiation was taken when a leaf primordium was visible (∼1 mm). For example: two new leaves at day 8 equates to a rate of leaf production per day, from day 1, of 0.25. Flowering time was taken when a bolting stem became visible (∼1 mm in length) at the center of the rosette. The average RLP was calculated for the WT and *ext18* plant populations, throughout vegetative development (from initiation of first true leaf to bolting) at each consecutive time interval by dividing the number of leaf primordia that appeared in that interval by the corresponding number of days. The PL measured the time between the initiations of two successive leaf primordia (days per leaf). The *t*-test determined significance.

### Plasmid Construction and Plant Transformation

For the purpose of testing if the WT *EXT18* gene could rescue the *ext18* mutant phenotype, a 3.78 kb *Eco*RI to *Xho*I DNA fragment from WT *Arabidopsis*, which included the putative *EXT18* gene with 1,773 bp up-stream and 679 bp downstream of the amino acid coding region, was ligated into the *Eco*RI and *Sal*I sites of the pCAMBIA1300 binary vector (Cambia, GPO Box 3200, Canberra 2601, Australia) for plant transformation. The 3.78 kbp (kilobase pair) fragment was prepared by PCR amplification from BACF28B23 [from the *Arabidopsis* Biological Resource Center (ABRC), Ohio State University, Columbus, OH 43210, USA] using primers #603bf and #604br (Supplementary Table S3), followed by restriction digestion with *Eco*RI and *Xho*I. This *EXT18*-pCAMBIA plasmid, named pPS3, was mobilized (Holster et al., 1978; Hofgen and Willmitzer, 1988) from *E. coli* into *Agrobacterium tumefaciens* strain EHA105 (Hood et al., 1993) for delivery to homozygous *ext18* plants (T_0_) using the floral dip method (Clough and Bent, 1998). Transformants (T_1_) were selected on half-MS medium containing Hygromycin 20 mg per L (Hyg^20^), and confirmed by segregation of T2 seedlings in the presence of Hyg^20^ and/or km^40^. Presence of the transgene and its zygosity were identified by PCR. Independently transformed lines of homozygous *ext18* were selected, and tested for *EXT18* gene expression (see below).

### Microscopy and Staining

For bright field observations, a SMZ-U stereoscopic microscope (Nikon, Tokyo, Japan) was used, with a mounted SPOT Insight CCD^TM^ (Diagnostic Instruments, Inc., Sterling Heights, MI, USA). For bright field with higher magnification or for fluorescence, a Labophot-2 microscope (Nikon) was used, with a fluorescence source and appropriate filters, and a mounted SPOT cooled CCD (Diagnostic Instruments, Inc.). Mature anthers in open flowers were mounted in 80% glycerol (v/v in water) solution on glass slides for observation. Callose in pollen tubes was stained with decolorized aniline blue (DAB) at 0.1% (w/v in water; Smith and McCully, 1978).

#### DAPI Staining

Newly opened flowers were harvested in 500 μL pollen isolation buffer (100 mM Na_2_PO_4_ buffer, pH 7.0, 1 mM EDTA and 0.1% (v/v) Triton X-100), briefly vortexed to release pollen and centrifuged for 1 min at 1,500 *g* to pellet the pollen, followed by resuspension in 20 μL pollen isolation buffer containing DAPI (4′,6-diamidino-2-phenylindole, dihydrochloride, Molecular Probes) at 1 mg/l. Samples were kept at 4°C overnight, before drops were placed on glass slides under coverslips; nuclei were viewed using a fluorescence microscope with a DAPI filter set.

#### I_2_–KI Staining

Pollen was harvested from freshly opened flowers in a few drops of I_2_–KI (Lugol solution [catalog # 62650], from Sigma-Aldrich, St. Louis, MO, USA); after 10 min the pollen was pelleted by briefly centrifuging, washed in distilled water, and mounted on glass slides in 80% glycerol for microscopic observed and image capture.

#### Alexander Staining

Inflorescences were collected from adult plants and fixed in ECA (100% ethanol: chloroform: acetic acid, ratio 6:3:1, respectively) for 1–3 h, followed by incubation in a 1:50 dilution (in water) of Alexander’s Stain (Alexander, 1969) at 65°C for 7 h. Flowers were then washed in 10% glycerol before the anthers were dissected, and mounted on slides in 80% glycerol for microscopic observation and image capture.

#### *In Vivo* Pollen Germination

Anthers were removed at anthesis and used to brush pollen on stigmas to complete coverage. Pistils were dissected at 5 h after pollination and fixed in 3:1 ethanol:acetic acid (v/v) for 30 min, followed by softening in 1 M NaOH overnight at room temperature, washing x3 with sterile H_2_O, and then stained with 0.1% Aniline Blue (w/v in 0.1 M K_3_PO_4_ buffer pH 8.5) for more than 2 h in complete darkness. Pistils were briefly rinsed in 0.1 M K_3_PO_4_ buffer, mounted in 80% glycerol and viewed under UV. Method is based on (Kho and Baer, 1968).

#### *In Vitro* Pollen Germination

Pollen from freshly opened flowers was placed on germination medium (20% [w/v] sucrose, 0.07% CaCl_2_ and 0.01% H_3_BO_3_, 0.5% [w/v] low temp melting agarose), which had been freshly solidified as a flat pad to form a germination platform, and placed in a moisture chamber for 10 h at room temp. Method is based on (Boavida and McCormick, 2007). Pollen germination was viewed using a LABOPHOT-2 in bright field and germination counts were performed manually.

#### GUS Reporter Gene Localization

Wild type, homozygous and heterozygous *ext18* seedlings and plant parts were harvested in 90% acetone (v/v) on ice prior to doing the histochemical assay for GUS activity (Jefferson et al., 1987; Li et al., 2008).

### Gene Expression Analysis by Quantitative Reverse Transcription-PCR (qRT-PCR)

Seedlings (12 DAG) were harvested in liquid nitrogen. Total RNA isolation, cDNA synthesis and qRT-PCR analysis, using Power SYBR Green PCR master mix (Applied Biosystems, Foster City, CA, USA) in an ABI 7900 HT Fast Real-Time PCR system (Applied Biosystems), were all performed as previously described (Saha et al., 2013). Gene expression was normalized using *EIF4a-2* as the reference gene, and relative gene expression was calculated as the mean of three biological replicates and three technical replicates.

### Primer Design, EXT Sequences, and their Analysis

Primer design is as previously described (Saha et al., 2013). *EXT* sequences were version TAIR10 (The *Arabidopsis* Information Resource, Phoenix Bioinformatics, Redwood City, CA 94063, USA). All of the *EXT* sequence primer pairs used were designed, and confirmed as specific for their individual *EXT* gene. Sequence comparisons were done using the Lipman–Pearson method (Ktuple, 2; Gap Penalty 4; Gap Length penalty, 12) in MegAlign (DNASTAR, Inc., Madison, WI, USA). Translated EXTs and signal peptides (Bendtsen et al., 2004) were predicted from DNA sequence analysis.

## Conflict of Interest Statement

The authors declare that the research was conducted in the absence of any commercial or financial relationships that could be construed as a potential conflict of interest.
