# Supplementary material for: EXTENSIN18 is required for full male fertility as well as normal vegetative growth in Arabidopsis
Source: Front Plant Sci. 2015 Jul 22;6:553. doi: 10.3389/fpls.2015.00553 (PMC4510346; doi:10.3389/fpls.2015.00553)
Supplement: Supplementary file 1 [file Data_Sheet_1.PDF]

Supplementary Material

EXTENSIN18 is required for full male fertility as well as normal vegetative growth in *Arabidopsis*

Pratibha Choudhary[1,†], Prasenjit Saha[1,†], Tui Ray[2,†], Yuhong Tang[2], David Yang[1], and Maura C. Cannon[1*]

[1] Department of Biochemistry & Molecular Biology, University of Massachusetts, 710 North Pleasant Street, Massachusetts 01003, U.S.A.
[2] Plant Biology Division, The Samuel Roberts Noble Foundation, Ardmore, Oklahoma 73401, U.S.A.

***Correspondence:**
Maura C Cannon, Department of Biochemistry & Molecular Biology, University of Massachusetts, 710 North Pleasant Street, Amherst, MA 01003, U.S.A.
mcannon@biochem.umass.edu

[†]**Present addresses:**
Pratibha Choudhary, Department of Biology, Syracuse University, Syracuse, NY 13244, U.S.A.
Prasenjit Saha, Department of Plant Sciences, University of California, Davis, CA 95616, U.S.A.
Tui Ray, Department of Plant Sciences, University of California, Davis, CA 95616, U.S.A.

Reference for Supplementary Data

Bendtsen, J.D., Nielsen, H., Engelbrecht, J., von Heijne, G., and Brunak, S. (2004). Improved prediction of signal peptides: SignalP 3.0. J Mol Biol. **340**:783-795.

MANPNGWPSLLMLVIALYSVSAHTSA/
QYTYSPPSPPSYVYKPPTHIYS
SPPPPPYVYS
SPPPPPYIYK
SPPPPPYVYS
SPPPPPYIYK
SPPPPPYVYS
SPPPPPYIYK
SPPPPPYVYS
SPPPPPYVYK
SPPPPPYVYN
SPPPPPYVYK
SPPPPPYVYS
SPPPPPYVYK
SPPPPPYVYS
SPPPPPYVYK
SPPPPPYVYS
SPPPPPYVYK
SPPPPPYVYS
SPPPPPYVYK
SPPPPPYVYS
SPPPPPYVYK
SPPPPPYVYS
SPPPPPYVYK
SPPPPPYVYS
SPPPPPYVYK
SPPPPPYVYS
SPPPPPYVYK
SPPPPPYVYS
SPPPPPYVYK
SPPPPPYVYN
SPPPPPYVYK
SPPPPPYVYS
SPPPSPYVYK
SPPPPPYVYS
SPPPPPYVYK
SPPPPPYVYS
SPPPPPYVYK
SPPPPPYVYS
SPPPPPYVYK
SPPPPPYVYS
SPPPPPYVYK
SPSPPPYVYK
SPPPPPSYSY
SYSSPPPPIY

Supplemental Figure 1: Predicted amino acid sequence of WT non-hydroxylated pre-EXT19 (At1g26240). The sequence is arranged to emphasize the 10-residue major repetitive motif (MRM), S(P)$_5$YVYK/S. Like most classical EXTs, EXT18 has an N-terminal sequence and a C-terminal sequence containing mainly the same amino acid residues found in the MRM but not exactly in register. The terminal residue is Y, as is the case with 19 of the 20 Arabidopsis network-forming type EXTs. EXT19 is classified as Group 2a EXT because it has a high abundance of YXY (44 YXY) motifs, low abundance of SPSP motifs, and no tri-C motif. /, indicates cleavage site of the 26 residue predicted signal peptide (Bendtsen et al., 2004). The mature EXT19 has 452 residues. YELLOW, highlights putative Isodityrosine (Idt) motifs, lone Y, and 2Y; GREEN highlights positively charged amino acids; MAGENTA highlights the SPSP motif.

Compare sequence with EXT18 (Figure 1), also a Group 2a EXT.

MGAPMASFLVLAFSLAFVSQTTA/
NYFYS
SPPPPVKHY
SPPPVYK
SPPPPVKHY
SPPPVYK
SPPPPVKHY
SPPPVYK
SPPPPVKYY
SPPPVYK
SPPPPVYK
SPPPPVKHY
SPPPVYK
SPPPPVKHY
SPPPVYK
SPPPPVKHY
SPPPVYK
SPPPPVKHY
SPPPVYK
SPPPPVKYY
SPPPVYK
SPPPPVKHY
SPPPVYK
SPPPPVKYY
SPPPVYK
SPPPPVKHY
SPPPVYK
SPPPPVKYY
SPPPVYK
SPPPPVHY
SPPPVVYH
SPPPPVHY
SPPPVVYH
SPPPPVHY
SPPPVVYH
SPPPPVHY
SPPPVVYH
SPPPPVHY
SPPPVVYH
SPPPPKKHYEYK
SPPPPVHY
SPPTVYH
SPPPPVHHY
SPPHQPYLYK
SPPPPHY

Supplemental Figure 2. Predicted amino acid sequence of WT non-hydroxylated pre-EXT1 (At1g76930). The sequence is arranged to emphasize the 7-9-residue major repetitive motif (MRM). The terminal residue is Y, as is the case with 19 of the 20 Arabidopsis network-forming type EXTs. EXT1 is classified as a Group 1 EXT as it has only 3 YXY motifs, and does not have an SPSP nor tri-C motif.
/, indicates cleavage site of the 23 residue predicted signal peptide (Bendtsen et al., 2004). The mature EXT1 has 454 residues.
YELLOW, highlights putative Isodityrosine (Idt) motifs, lone Y, and 2Y; GREEN highlights positively charged amino acids; MAGENTA highlights the SPSP motif.

Compare sequence with EXT18 (Figure 1), and EXT19 (above), also Group 2a EXTs, unlike EXT1.

```
MGSPMASLVATLLVLTISLTFVSQSTA/
NYFYSSPPPPVK
HYTPPVKHYSPPPVYHSPPPPKKHYEYK
SPPPPVKHYSPPPVYHSPPPPKKHYVYK
SPPPPVKHYSPPPVYHSPPPPKKHYVYK
SPPPPVKHYSPPPVYHSPPPPKKHYVYK
SPPPPVKHYSPPPVYHSPPPPKKHYVYK
SPPPPVKHYSPPPVYHSPPPPKKHYVYK
SPPPPVKHYSPPPVYHSPPPPKKHYVYK
SPPPPVKHYSPPPVYHSPPPPKKHYVYK
SPPPPVKHYSPPPVYHSPPPPKKHYVYK
SPPPPVKHYSPPPVYHSPPPPKKHYVYK
SPPPPVKHYSPPPVYHSPPPPKKHYVYK
SPPPPVKHYSPPPVYHSPPPPKEKYVYK
SPPPPVKHYSPPPVYHSPPPPKKHYVYK
SPPPPPPVHHYSPPHHPYLYKSPPPPYHY
```

Supplemental Figure 3. Amino acid sequence of WT non-hydroxylated pre-EXT3 (At1g21310). The sequence is arranged to emphasize the 28-residue major repetitive motif (MRM), each containing a YXY motif. Like other classical EXTs, EXT3 has an N-terminal sequence and a C-terminal sequence containing mainly the same amino acid residues found in the MRM but not exactly in register. The terminal residue is Y, as is the case with 19 of the 20 Arabidopsis network-forming type EXTs. EXT3 is classified as Type 2a EXT because it has an abundance of YXY (16 YXY) motifs, and no SPSP nor tri-C motifs.
/, indicates cleavage site of the 27 residue predicted signal peptide (Bendtsen et al., 2004). The mature EXT3 has 404 residues.
YELLOW, highlights putative Isodityrosine (Idt) motifs, and lone Y residues. Note the high abundance of lone Y residues compared to EXT18, and EXT19. GREEN, highlights positively charged amino acids.

Compare with EXT18 (Figure 1) and EXT19 (above), which are also Group 2a EXTs.

Supplemental Table 1: Ovule counts per silique following reciprocal crosses of WT with the *ext18* GT8324 mutant.

Female-WT crossed with male *ext18*				Female *ext18* crossed with male WT			
Silique #	Fertilized ovules	Un-fertilized Ovules	Total	Silique #	Fertilized ovules	Un-fertilized Ovules	Total
1	5	15	20	1	36	2	38
2	6	17	23	2	34	2	36
3	8	11	19	3	39	1	40
4	2	16	18	4	40	2	42
5	7	11	18	5	38	3	41
6	5	10	15	6	35	2	37
7	8	14	22	7	38	1	39
8	5	19	24	8	42	2	44
9	3	13	16	9	39	1	40
10	3	16	19	10	38	2	40
Avg	5.2	14.2	19.4	Ave	37.9	1.8	39.7
SD	2.10	2.94	2.91	SD	2.38	0.63	2.36

Supplemental Table 2: Fold change (Log_2) in expression of classical *EXT* genes in seedlings of *ext18* GT8324 mutant, and in three independently transformed *ext18*-GT8324 lines carrying a transgene of *EXT18* (TL1, TL2, and TL3), compared to that of WT.

			ext18 vs WT		*ext18*-TL1 vs WT		*ext18*-TL2 vs WT		*ext18*-TL3 vs WT	
EXT Groups[a]: features	Name	Gene ID	Log_2	*P*-value	Log_2	*P*-value	Log_2	*P*-value	Log_2	*P*-value
Gp 1: Idt-poor	EXT1	At1g76930	1.65	0.000	1.47	0.001	1.49	0.005	1.16	0.004
	EXT23	At5g19810	0.43	0.140	-0.03	0.955	0.23	0.397	0.19	0.476
Gp 2a: Idt-rich	EXT3	At1g21310	-0.62	0.036	-0.19	0.082	-0.28	0.133	-0.04	0.821
	EXT18	At1g26250	nv	nv	-0.78	0.372	-0.20	0.798	-0.43	0.597
	EXT19	At1g26240	3.05	0.006	1.33	0.364	0.93	0.172	0.77	0.239
	EXT20	At4g08370	2.53	0.113	2.09	0.028	1.44	0.037	2.39	0.004
	EXT21	At2g43150	-0.20	0.462	0.05	0.797	0.17	0.389	0.21	0.368
	EXT22	At4g08380	-0.23	0.851	2.52	0.112	2.05	0.101	1.49	0.064
Gp 2b: Idt-rich, SPSP motifs	EXT6	At2g24980	-0.85	0.050	-3.42	0.001	-2.73	0.001	-2.03	0.002
	EXT7	At4g08400	-0.88	0.106	-2.68	0.009	-2.53	0.009	-2.04	0.012
	EXT8	At4g08410	-1.23	0.003	-4.28	0.000	-3.82	0.000	-2.57	0.000
	EXT9	At5g06630	-0.72	0.046	-4.06	0.000	-3.08	0.000	-2.21	0.001
	EXT10	At5g06640	-1.53	0.004	-4.05	0.001	-3.04	0.001	-2.74	0.001
	EXT11	At5g49080	0.33	0.060	1.31	0.002	0.66	0.154	0.27	0.266
	EXT12	At4g13390	-1.64	0.018	-3.55	0.006	-2.90	0.006	-2.24	0.009
	EXT13	At5g35190	-1.35	0.035	-4.60	0.005	-3.52	0.006	-2.55	0.008
Gp 2c: Idt-rich, SPSP motifs, 1 tri-C motif	EXT2	At3g54590	-1.24	0.006	-3.52	0.000	-2.63	0.000	-2.17	0.001
	EXT15	At1g23720	-0.44	0.227	-1.90	0.009	-1.46	0.010	-0.94	0.032
	EXT16	At3g28550	-1.30	0.007	-3.11	0.000	-2.27	0.001	-1.64	0.001
	EXT17	At3g54580	-1.50	0.019	-4.20	0.004	-2.88	0.006	-2.36	0.008

[a], EXT Group Classification, [see Cannon et al. (2008) Table S1].
Grey shading = not significant at ($P > 0.05$).
nv = no value (no detectable expression of EXT18).

Supplemental Table 3. Primers used for qRT-PCR (rows 1-21), and PCR (last eight rows).

Primer Name / #	Gene ID	Forward (f) sequence (5'-3')	Reverse (r) sequence (5'-3')
EXT1	At1g76930	ACAAATCACCACCTCCTCCTCACT	TCTCCCGTCAACGATCTTGTGTCT
EXT23	At5g19810	TTTCTCCTCCTCCACCCACAGTTA	ATCTCTTGTAGGAACGAGCGGCTT
EXT3	At1g21310	GGCCTCTTTAGTGGCAACTTTGCTTG	GTGGTGGAGGAGAAGAATAGAAATAGT
EXT18	At1g26250	GGCCAGTCCTAATTGGCCATCTTT	TGTAAACATATGGCGGAAGCGGTG
EXT19	At1g26240	CCAATCCTAATGGTTGGCCGTCTT	ATGATGGTGGTGATGGTGGAGAGT
EXT20	At4g08370	GCCTTCTATGTCGTTGTAGTGCCT	AGACATATGGTTGTGGTGGTGGTG
EXT21	At2g43150	TCATGCCAAGGCTCAATGGGTAGT	ACTTAACCGGAGGTGGTGGTGATT
EXT22	At4g08380	GTTGCTGCGCATACAAGTGCTCAA	AGGCGTATGGTGGTGGTGAACTAT
EXT6	At2g24980	CCTCAAACCCCAAGTTACAATTCT	TAGGGCCTTTGTGTTCATAGCTGG
EXT7	At4g08400	TAACTCCCCACCCCCACCATAT	TGGTGGAGTTTTGTAGTTTACC
EXT8	At4g08410	CCAAAACAAAGAAAAAAGATAGTTGC	AATGGCACTTAGGACGACAACA
EXT9	At5g06630	TGGTCCTAAGTGCTATTGCGGCTA	GCGTACTTTGGGCCTTTGTGTTCA
EXT10	At5g06640	GGCATTGCATGGTTTACGTTGTGG	TGGCTGGGAGAATTGTAATGTGGG
EXT11	At5g49080	AGTCGTCATCCACCAATCACCGTA	CACCACAACTTTGTTAGTACTTAAGCTGG
EXT12	At4g13390	ATTGCCTAGTTTATGTCGTGGTCTT	GAGGGTGAGTCGTATGCTGTCA
EXT13	At5g35190	GGAGCGAGTGCATTGCATGGTTTA	AGGTAGTGGTGATGCCGAGTAGTA
EXT2	At3g54590	TCTCCGCCACCGTTGTATTCTTCA	ATTCCACTTCAGGAGCTGGTGTGT
EXT15	At1g23720	TTGCTGCGTCTTATGAGCCCTACA	ACCTTGGGAGATGGCGAATACGAT
EXT16	At3g28550	TGCGGCATATGAGCCATACACAGA	TAGTGGTGGTGGTGGAGAACTGTA
EXT17	At3g54580	ACCCATCTCATTTGCGCTCTAGGA	ACTTCAGGAGCCGGTGAGTAAGTT
EIF4A-2	At1g54270	GGCTGAATGAAGTTCTCGATGGACAG	ACGAGAGCCTGGCACTGGAGAAG
158f & 113r	At1g21310	GTTTCCCCTTTTAACTTAGAGGCC	CACACTCGTTGTAAAATGGTAACTTACG
158f & 114r		GTTTCCCCTTTTAACTTAGAGGCC	CCAACGCTGATCAATTCCAC
12f & 14r	At1g26250	ACCATACCAGTTGCAGTGATGAC	TGGTGGAGGAGGCGACTTGTAGAC
12f & 114r		ACCATACCAGTTGCAGTGATGAC	CCAACGCTGATCAATTCCAC
17f & 19r	At1g26250	TACAAATCTCCACCACCGCCTC	TGACAACGTCTCGAAAGGGAAAGC
17f & 114r		TACAAATCTCCACCACCGCCTC	CCAACGCTGATCAATTCCAC
603bf & 604br	At1g26250	AAAAAACTCGAGCTTGAAAGAAGAAATAGCTC	GAAGACAAGCCCACCTAAAC
539f & 540r		AAGACCTGCCTGAAACCGAAC	AAGAAGATGTTGGCGACCTCG
